# Expression of Lysine-specific demethylase 1 in human epithelial ovarian cancer

**DOI:** 10.1186/s13048-015-0155-1

**Published:** 2015-05-09

**Authors:** Cong Chen, Jing Ge, Qibin Lu, Guoqiang Ping, Chunqing Yang, Xuefeng Fang

**Affiliations:** Department of Gynecology of Traditional Chinese Medicine, Jiangsu Provincial Hospital of Traditional Chinese Medicine Affiliated to Nanjing University of Traditional Chinese Medicine, Nanjing, Jiangsu 210029 China; Department of Endocrinology, Jiangsu Provincial Hospital of Traditional Chinese Medicine Affiliated to Nanjing University of Traditional Chinese Medicine, Nanjing, Jiangsu Province 210029 China; Department of Pathology, First Affiliated Hospital of Nanjing Medical University, Nanjing, Jiangsu 210029 China; Department of Medical Oncology, Second Affiliated Hospital, Zhejiang University College of Medicine, Hangzhou, Zhejiang 310009 China

**Keywords:** Epithelial ovarian cancer, Lysine-specific demethylase 1, Serous cystadenocarcinoma, Mucinous cystadenocarcinoma

## Abstract

**Background:**

Lysine-specific demethylase 1(LSD1) is implicated in the tumorigenesis and progression in various cancers. However, the expression of LSD1 in epithelial ovarian cancer and its clinical significance has not been examined in detail.

**Methods:**

Immunohistochemical was used to detect the expression of LSD1 in normal ovarian epithelial tissues, cystadenoma, borderline cystadenoma, and cystadenocarcinoma. Next, the correlations between expression of LSD1 and clinicopathological features was assessed in 96 species of serous cystadenocarcinoma and 36 species of mucinous cystadenocarcinoma.

**Results:**

Immunohistochemical results showed that the expression of LSD1 was gradually increased from benign cystadenoma and borderline cystadenoma to cystadenocarcinoma. The positive ratio of LSD1 expression was 50% in normal ovarian epithelial tissues, 72% in serous cystadenoma, 73% in mucinous cystadenoma, 82% in borderline serous cystadenoma, 83% in borderline mucinous cystadenoma, 94% in serous cystadenocarcinoma and 92% in mucinous cystadenocarcinoma, respectively. LSD1 expression levels were associated with International Federation of Gynecology and Obstetrics stage and lymphatic metastasis in both serous and mucinous cystadenocarcinoma samples. Kaplan-Meier curves suggested that overall survival time of patients with high LSD1 expression was significantly shorter than that of patients with low LSD1 expression. Multivariate Cox proportional hazard regression indicated that higher LSD1 expression was a significant independent predictor of poor survival of EOC patients (*P* = 0.016).

**Conclusions:**

These results suggest that LSD1 may be involved in carcinogenesis and progression with promising therapeutic potential for epithelial ovarian cancer.

## Introduction

Ovarian cancer is the second most common gynecologic cancer and ranks the most lethal gynecological malignancy in the world, due to its high incidence of metastasis and high relapse rate [1]. The vast majority of ovarian cancer will be epithelial ovarian cancer (EOC), which comprises three major histological subtypes (serous, mucinous, and endometrioid). Unfortunately, the most of EOC patients were diagnosed at FIGO (International Federation of Gynecology and Obstetrics stage) III/IV stage and had unfavorable prognosis, with a frustrating 5-year overall survival (<50%) [2]. Although the molecular alterations in EOC have been widely studied, studying the mechanism that regulates the initiation and progression will provide further insights into the development and progression of EOC.

Lysine-specific demethylase 1 (LSD1) is the first histone demethylase to be discovered [3]. Overexpression of LSD1 has also been associated with unfavourable clinicopathological characteristics and poor prognosis in numerous tumors, such as hepatocellular carcinoma, colon cancer, breast cancer, prostate cancer, and non-small cell lung cancer [4-8]. Recently, one study showed that the expression of LSD1 mRNA was increased in ovarian tumors and LSD1 mRNA was overexpressed in stage IIIC and high-grade ovarian tumors [9]. However, the expression and significance of LSD1 protein in EOC is still poorly understood.

This study was to systematically investigate LSD1 protein expression in normal ovarian epithelium, benign cystadenoma, borderline cystadenoma, and cystadenocarcinoma using immunohistochemical (IHC) staining. The potential correlation between the expression level of LSD1 and clinicopathological features of EOC was also analyzed.

## Materials and methods

### Tissue specimens

The specimens (n = 407) were randomly obtained from patients with complete clinical data in the Pathological Department of Jiangsu Province Hospital and Second Affiliated Hospital of Zhejiang University College of Medicine between March 2000 and May 2013. All patients were evaluated for histological type and graded by the two gynecological pathologists. The samples consisted of 50 normal ovarian epithelia species; 199 species of serous epithelial lesions, and 158 species of mucinous epithelial lesions. None of the patients were treated with chemotherapy, immunotherapy or radiotherapy prior to specimen collection. Tissue samples were obtained after patients’ written informed consent under a general tissue collection protocol approved by The Research Ethics Committee of Jiangsu Province Hospital and The Research Ethics Committee of Second Affiliated Hospital of Zhejiang University College of Medicine.

### Immunohistochemistry

The immunohistochemistry (IHC) for LSD1 (1:100; Cell Signaling Inc., Danvers, MA, USA) was performed on formalin-fixed, paraffin-embedded tissue sections using steam heatinduced epitope retrieval and the DAB chromogen (Boster, Wuhan, China). Positive cells were indicated by the presence of brown staining in the nucleus. The LSD1 expression was evaluated based on intensity of staining and distribution of positive cells. Both the percentage of positive cells and the staining intensity were evaluated under double-blind conditions. The percentage positivity was scored as four classes: “0” (<5%, negative), “1” (5–25%, sporadic), “2” (25–50%, focal), or “3” (>50%, diffuse). The staining intensity was scored as “0” (no staining), “1” (weakly stained), “2” (moderately stained), or “3” (strongly stained). The LSD1 immunostaining score was calculated as the percentage positive score × the staining intensity score and ranged as 0, 1, 2, 3, 4, 6, and 9. The sum-indexes (−), (+), (++), and (+++) indicated overall staining score of 0, 1–3, 4–6, and 9, respectively. For statistical analysis, sum-indexes (−) and (+) were defined as low LSD1 expression, while sum-indexes (++) and (+++) were defined as high LSD1 expression.

### Statistical analysis

Statistical analysis was carried out using SPSS version 16.0. LSD1 expression in different groups was analyzed using the non-parametric tests. The approximate normal distribution of the two groups is represented as Z-score. The correlation between LSD1 expression and the clinicopathological features was assessed by chi-square test. Survival analyses were performed using the log-rank test and Kaplan-Meier plots approach. Variables with *P* value < 0.05 in univariate analysis were used in thesubsequent multivariate analysis on a basis of Cox proportionalhazards model. For all analyses, the level of significance was set at *P* < 0.05.

## Results

### Expression of LSD1 in ovarian epithelial lesions

**Figure 1:**
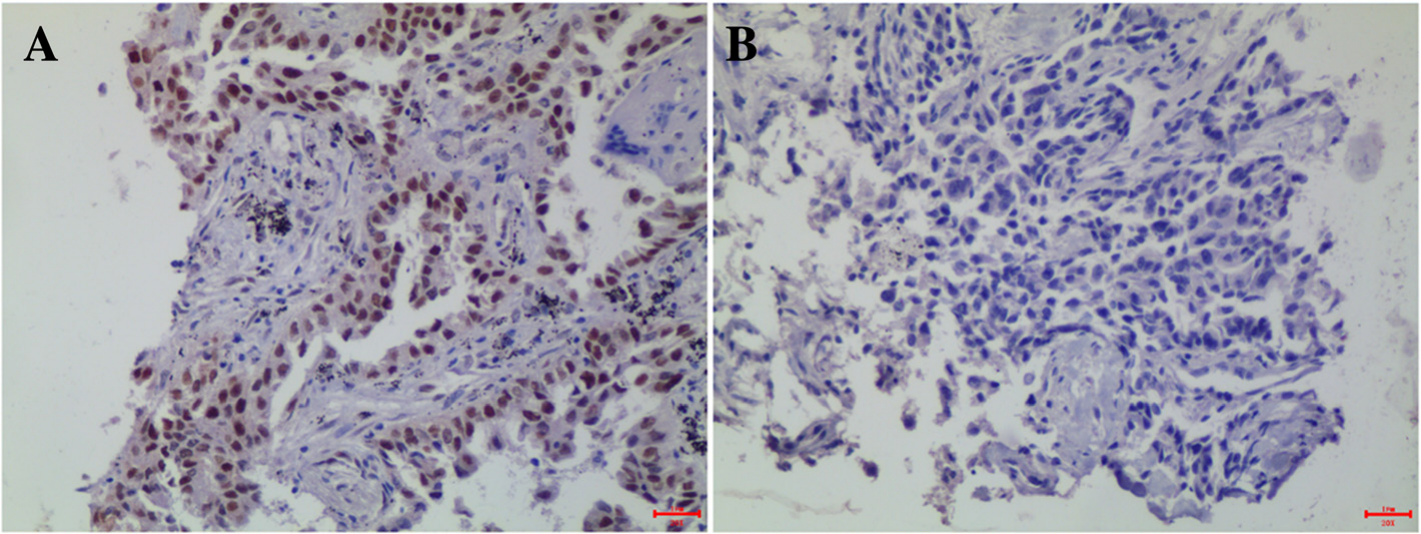
LSD1 expression in positive control and negtive control. **(A)** A lung adenocarcinomas that overexpression of LSD1 was as uesed as positive control. **(B)** “No primary antibody” including everything but the primary antibody showed negtive stain of LSD1 in the same lung adenocarcinomas tissue sample.

**Figure 2:**
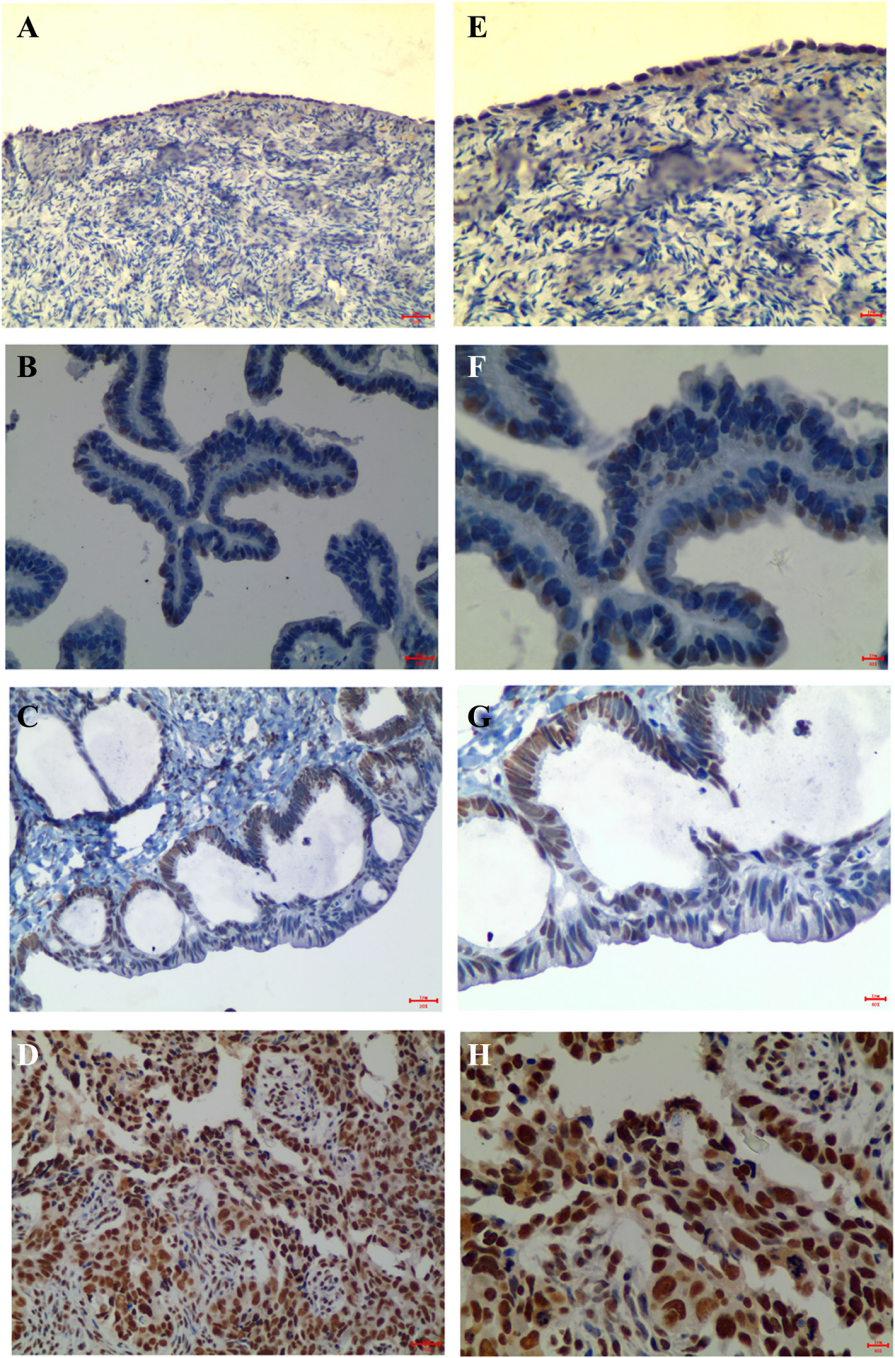
LSD1 expression and localization in **(A)** normal ovarian epithelia, **(B)** serous cystadenoma, **(C)** borderline serous cystadenoma, and **(D)** serous cystadenocarcinoma (20×, *bar* = 100 μm). The Higher magnification (40×, *bar* = 1 μm) was shown as the right image **(E, G, F, H)**.

**Figure 3:**
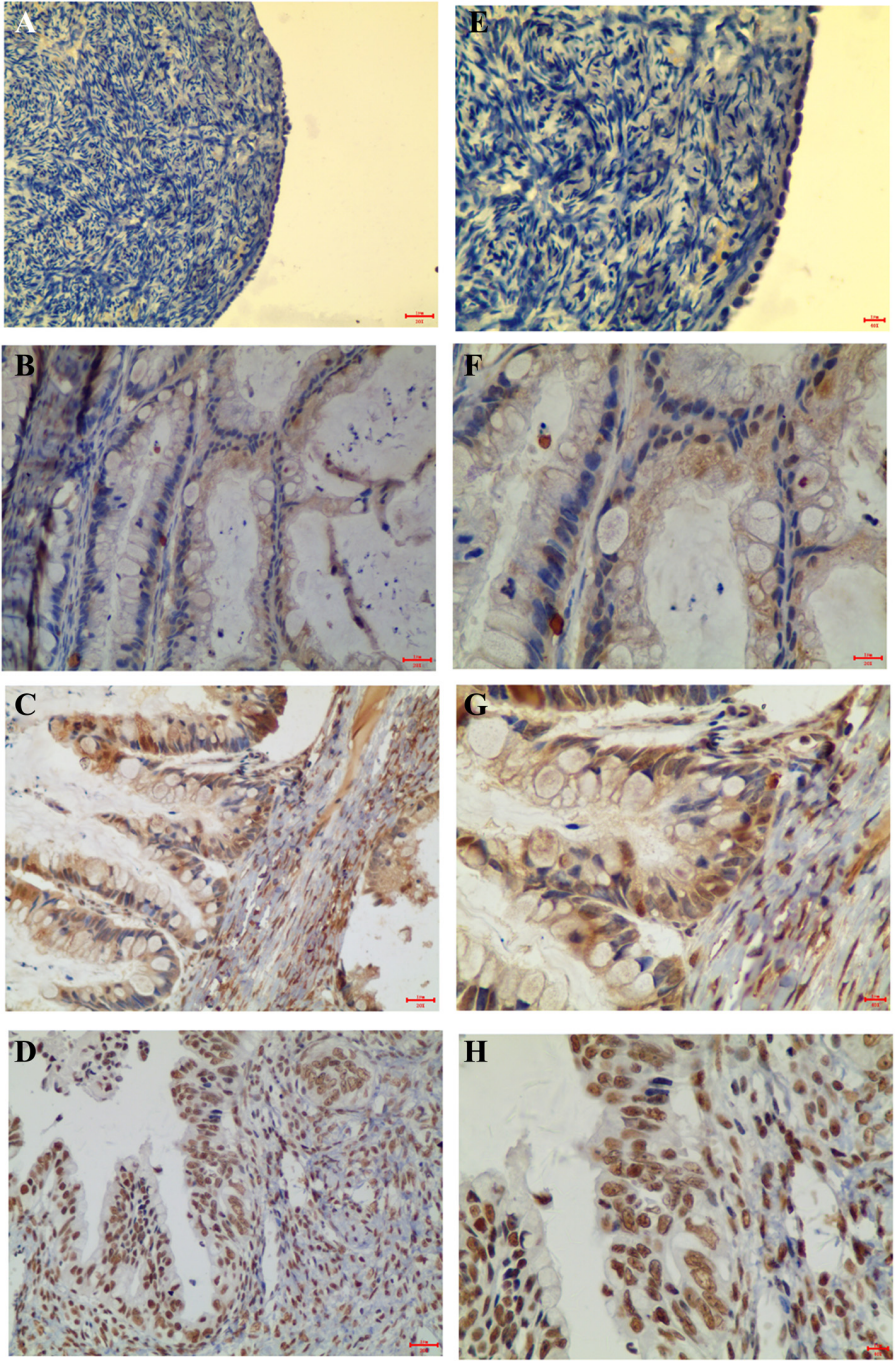
LSD1 expression and localization in **(A)** normal ovarian epithelia, **(B)** mucinous cystadenoma, **(C)** borderline mucinous cystadenoma, and **(D)** mucinous cystadenocarcinoma (20×, *bar* = 100 μm). The Higher magnification (40×, *bar* = 1 μm) was shown as the right image **(E, G, F, H)**.

A lung adenocarcinomas that overexpression of LSD1 was as uesed as positive control as shown in Figure 1A. “No primary antibody” control showed negative stain of LSD1 in the same lung adenocarcinomas tissue sample (Figure 1B). IHC staining revealed that LSD1 was localized mainly to the cell nucleus (dark brown nuclei) in different ovarian epithelial lesions (Figures 2 and 3). Positive IHC staining of LSD1 was observed in 50% of normal ovarian epithelia, 72% of serous cystadenoma, 73% of mucinous cystadenoma, 82% of borderline serous and mucinous cystadenoma, 94% of serous cystadenocarcinoma, and 92% of mucinous cystadenocarcinoma, respectively (Table 1). LSD1 expression was significant statistical difference between the normal ovarian epithelia and all of carcinomas (benign, borderline tumors and carcinomas; P < 0.001). In additional, the incidence of LSD1 detected in epithelial carcinomas was significantly higher than that in benign and borderline tumors, both in subtypes of serous and mucinous (P < 0.001). The incidence of LSD1 detected was gradually increased from benign and borderline to malignant ovarian tumors, suggusting that LSD1 protein was up-regulated in the development of serous or mucinous ovarian epithelial carcinoma.

**Table 1:** **Expression of LSD1 in different ovarian epithelial lesions and normal ovarian epithelia**

**Clinicopathological features**		**Cases (N)**	**LSD1**				**Positive (%)**	**P**
			**—**	**+**	**++**	**+++**		
Normal ovarian epithelia		50	25	20	5	0	50	0.000^ab^
Serous epithelial lesions	Serous cystadenoma	53	15	25	10	3	72	0.010^c^
	Borderline serous cystadenoma	50	9	10	25	6	82	0.000^d^
								0.002^i^
	Serous cystadenocarcinoma	96	6	9	31	50	94	0.000^ejk^
Mucinous epithelial lesions	Mucinous cystadenoma	59	16	25	16	2	73	0.003^f^
	Borderline mucinous cystadenoma	63	11	17	29	6	83	0.000^g^
								0.009^l^
	Mucinous cystadenocarcinoma	36	3	5	9	19	92	0.000^hmn^

^a^Non-parametric Jonckheere-Terpstra test, P value of normal and serous groups.

^b^Non-parametric Jonckheere-Terpstra test, P value of normal and mucinous groups.

^c^normal ovarian epithelia versus serous cystadenoma; Z = −2.587, *P* = 0.010.

^d^normal ovarian epithelia versus borderline serous cystadenoma; Z = −5.068, *P* = 0.000.

^e^normal ovarian epithelia versus serous cystadenocarcinoma; Z = −8.404, *P* = 0.000.

^f^normal ovarian epithelia versus mucinous cystadenoma; Z = −3.000, *P* = 0.003.

^g^normal ovarian epithelia versus borderline mucinous cystadenoma; Z = −5.073, *P* = 0.000.

^h^normal ovarian epithelia versus mucinous cystadenocarcinoma; Z = −6.277, *P* = 0.000.

^i^serous cystadenoma versus borderline serous cystadenoma; Z = −3.126, *P* = 0.002.

^j^serous cystadenoma versus serous cystadenocarcinoma; Z = −7.106, *P* = 0.000.

^k^borderline serous cystadenoma versus serous cystadenocarcinoma; Z = −4.735, *P* = 0.000.

^l^mucinous cystadenoma versus borderline mucinous cystadenoma; Z = −2.611, *P* = 0.009.

^m^mucinous cystadenoma versus mucinous cystadenocarcinoma; Z = −5.146, *P* = 0.000.

^n^borderline mucinous cystadenoma versus mucinous cystadenocarcinoma; Z = −3.824, *P* = 0.000.

Z= Z-score.

### Association of LSD1 expression with clinicopathological features of EOC patient

The correlation between LSD1 expression in ovarian carcinomas detected by IHC and clinicopathological features was further analyzed, including age, FIGO stage, tumor grade, lymphatic metastasis status and peritoneal cytology. The results showed that LSD1 expression was correlated with FIGO stage (*P* = 0.006) and lymph node metastasis (*P* = 0.001) among serous tumors (Table 2). Among mucinous tumors, significant association was also found between LSD1 expression and FIGO stage (*P* = 0.037) and lymph node metastasis (*P* = 0.026).

**Table 2:** **Correlations between expression of LSD1 and clinicopathological features**

**Diagnosis**	**Characteristics**	**Cases (N)**	**IHC results of LSD1 (N)**		***p*** **-value**
			**Low**	**High**	
Serous cystadenocarcinoma	Age				0.352
	≦60	40	19	21	
	>60	56	30	26	
	FIGO stage				0.006
	I	7	4	3	
	II	23	5	18	
	III	54	4	50	
	IV	12	2	10	
	Tumor grade				0.707
	I	30	6	24	
	II	32	4	28	
	III	34	5	29	
	Lymphatic metastasis				0.001
	Negative	25	9	16	
	Positive	71	6	65	
	Peritoneal cytology				
	Negative	46	20	26	0.152
	Positive	50	30	20	
Mucinous cystadenocarcinoma	Age				0.179
	≦60	16	6	10	
	>60	20	13	7	
	FIGO stage				0.037
	I	6	4	2	
	II	9	3	6	
	III	12	1	11	
	IV	9	0	9	
	Tumor grade				0.995
	I	9	2	7	
	II	14	3	11	
	III	13	3	10	
	Lymphatic metastasis				0.026
	Negative	11	5	6	
	Positive	25	3	22	
	Peritoneal cytology				0.311
	Negative	15	9	6	
	Positive	21	8	13	

### Up-regulation of LSD1 is associated with poorer prognosis of EOC patient

**Figure 4:**
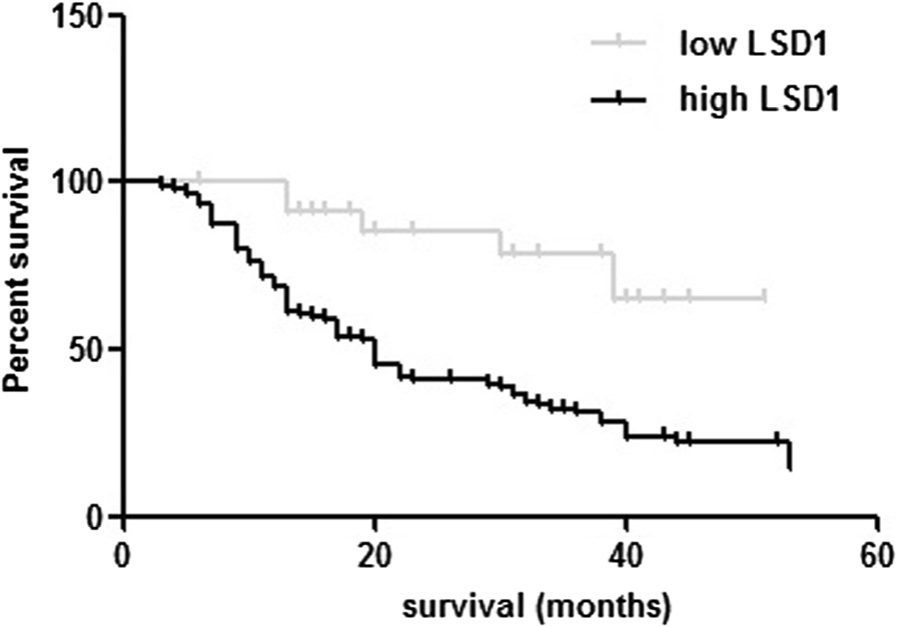
Overexpression of LSD1 predicts poor clinical outcome of EOC. Kaplan-Meier curves for overall survival according to expression of LSD1 in 132 EOC patients. (Log rank test, *P* = 0.0006).

The potential correlation between expression of LSD1 and EOC prognosis was addressed in the present study. Patients were classified into low LSD1 expression group and high LSD1 expression group according to the IHC results. Kaplan-Meier curves suggested that overall survival time of patients with high LSD1 expression was significantly shorter than that of patients with low LSD1 expression (*P* = 0.0006; Figure 4). Cox regression analyses were then conducted to analyze various prognostic parameters for survival of EOC patients. Univariate analysis identified three prognostic factors: FIGO stage (I, II, III, IV), lymphatic metastasis (negative, positive) and LSD1 expression (high, low). In multivariate Cox proportional hazard regression, LSD1 higher expression was a significant independent predictor of poor survival of EOC patients (*P* = 0.016), as well as FIGO stage (*P* = 0.019) (Table 3).

**Table 3:** **Univariate and multivariate Cox regression analyses of clinicopathologic characteristics for overall survival in EOC patients**

**Characteristic**		**Univariate analysis HR (95% CI)**	***P*** **value**	**Multivariate analysis HR (95% CI)**	***P*** **value**
Age (y)	≤60, >60	2.661(0.930-5.168)	0.169		
FIGO stage	I, II, III, IV	2.168(1.291-3.686)	0.001**	2 .265(0.919-5.899)	0.019*
Lymphatic metastasis	negative, positive	2.196(1.332-5.926)	0.016*	0.818(0.136-3.219)	0.669
Peritoneal cytology	negative, positive	1.566 (0.566-5.861)	0.519		
Tumor grade	I, II, III	1.265(0.661-2.256)	0.556		
LSD1 expression	low, high	2.233(1.129-5.688)	0.010*	2.808(1.131-6.967)	0.016*

HR, Hazard ratio; CI, Confidence interval. Statistical significance. *P < 0.05; **P < 0.01.

## Discussion

Epigenetic alteration by histone methylation or demethylation has been shown to play an essential role in carcinogenesis and cancer progression. The methylation status of histone demethylases plays an important role in the regulation of gene expression [10]. Inhibition of chromatin modifying enzymes such as histone demethylases is a potential therapeutic strategy to inhibit cancer growth [11]. LSD1 had been found to control gene expression by histone modification. LSD1 is described as the first identified histone demethylase which represses and activates transcription by specifically demethylating the methyl groups from mono- and di-methylated Lysine (Lys)4 of histone H3K4me and Lys9 of H3K9me [3,12]. Previous studies showed that LSD1 was aberrantly overexpressed in various kinds of cancer and higher expression of LSD1 associated with more aggression in breast cancer, prostate cancer, and lung cancer [5-7].

Only one study showed that overexpression of LSD1 mRNA in stage IIIC and high-grade ovarian tumors with the likely exception of mucinous tumors [9]. The higher levels of LSD1 mRNA was observed in serous, papillary serous, endometrioid, and clear cell tumors, but not in those of the mucinous subtype. The mucinous group in that cohort they studied consists of only a small number of tumors (n = 5). In our study, IHC results showed that higher expression of LSD1 protein in both serous and mucinous cystadenocarcinoma compared to normal ovarian tissue. We think this inconsistence is due to realtive larger number of mucinous cystadenocarcinoma (n = 36) detected in our study. In additional, diffrennt detection methods also contribute to the inconsistent results. Our results also showed that LSD1expression was increased with advanced FIGO stage, but not with tumor grade. High LSD1 expression was also associated with advanced tumor stage of pancreatic cancer but not tumor grade [13]. Our results indicated that high LSD1 expression was associated with more aggressive biological behavior. In fact, our results showed that LSD1 expression was correlated with lymph node metastasis among ovian tumors. There was also significant statistical difference of LSD1 protein expression between benign tumor groups and the normal groups. Therefore, LSD1 is potential to be an early diagnostic marker for serous or mucinous cystadenocarcinoma.

Furthermore, we found that the expression of LSD1 was gradually increased from benign and borderline tumors to epithelial carcinomas in a stepwise manner, both in subtypes of serous and mucinous, suggesting that LSD1 protein was up-regulated in the development of serous and mucinous ovarian epithelial carcinoma. So upregulation of LSD1 may be an early tumor promoting event in EOC. Previous reports also supported that LSD1 acts as an early tumor promoter in carcinogenesis through chromatin regulation [11,14-16]. LSD1 could repress p53 activity through demethylation of Lys370 in p53, thus inhibit p53-mediated apoptosis and contribute to carcinogenesis [14]. Upregulation of LSD1 has also been considered as an early tumor promoting event in breast carcinoma [15]. There is a gradual increase of LSD1 expression within tumor progression from pre-invasive ductal carcinoma in situ to invasive ductal breast carcinoma [15]. Interruption of LSD1 by using siRNA or chemical inhibitor could suppress cancer cell proliferation, migration and invasion in various cancers [7,17-20] LSD1 knockout colorectal cancer cells showed less tumorigenic both in vivo and in vitro [21]. Chemical LSD1 inhibition could cause cytotoxicity in ovarian cancer lines [9]. Thus, our results indicate that LSD1 may be involved in the carcinogenesis and progression of EOC. The concrete mechanism in which LSD1 is linked to EOC development is deserved to been further explored.

IHC results also showed that higher expression LSD1 was associated with FIGO stage or lymphatic metastasis in both ovarian serous cystadenocarcinoma and mucinous cystadenocarcinoma. Of note, we found that EOC patients with high LSD1 expression had shorter overall survival time than patients with low LSD1 expression. Importantly, the Cox regression analyses identified LSD1 expression as a novel predicting factor for overall survival of EOC patients. It indicates that LSD1 expression may serve as a novel prognostic marker for EOC patients, and high LSD1 expression may promote tumor metastasis and associate with poor survival in ovarian epithelial carcinoma patients.

In conclusion, our results indicate that LSD1 is involved in carcinogenesis and progression in EOC. LSD1 may be an early identification and prognosis marker of EOC with promising therapeutic potential.

## Abbreviations

LSD1: Lysine-specific demethylase 1
EOC: Epithelial ovarian cancer
IHC: Immunohistochemical
